# Adolescents’ experiences of acceptance and commitment therapy for depression: An interpretative phenomenological analysis of good-outcome cases

**DOI:** 10.3389/fpsyg.2023.1050227

**Published:** 2023-03-22

**Authors:** Jinping Ma, Lili Ji, Guohua Lu

**Affiliations:** ^1^School of Public Health, Weifang Medical University, Weifang, China; ^2^School of Nursing, Weifang Medical University, Weifang, China; ^3^School of Psychology, Weifang Medical University, Weifang, China

**Keywords:** adolescent, depression, acceptance and commitment therapy, treatment change, interpretative phenomenological analysis

## Abstract

**Introduction:**

Acceptance and commitment therapy (ACT) has been shown to help treat depression. However, little is known about the patient’s experiences with ACT. This study aimed to learn how it was used in adolescents with major depressive disorder who have achieved good treatment outcomes.

**Methods:**

Five adolescents with major depressive disorder with good treatment outcomes of ACT were enrolled in the semi-structured qualitative interview and analyzed using systematic textual condensation and interpretative phenomenological analysis.

**Results:**

Four primary themes emerged from the investigation. “Therapist relationships and characteristics” describes the therapist’s receptiveness and respect for adolescents with depression and having a trustworthy and sincere therapist. “Spaces to explore and experience” describes the ongoing process and content of acceptance of negative emotions and mindfulness practices in the healing process. “Do important things” refers to values and committed action. The “time settings” include the frequency and duration of treatment.

**Conclusion:**

Adolescents make positive changes with a receptive and respectful therapist by exploring themselves in a genuine and trusting therapeutic relationship. Improvement seems to come from being open to all thoughts and feelings and developing the ability to live in the present moment. Teenagers attach great importance to value-oriented behaviour. Therefore, treatment should target the critical areas of depressed adolescents to guide them towards recovery effectively.

## Background

Depressive disorder is a mental illness characterized by poor mood, loss of interest, pleasure, and various cognitive, somatic, and behavioral characteristics. Depression is a severe public health issue and one of the most common mental illnesses among children and adolescents. Depression in childhood and adolescence can disrupt social functioning, family relationships, and scholastic attainment, all of which damage career and social position, and major depression can lead to suicide (Sagatun et al., 2016; Clayborne et al., 2019).

Acceptance and Commitment Therapy (ACT) is one of the most representative experiential behavior therapies in the third wave of Cognitive and Behavior Therapy (CBT)(Hayes et al., 2013). Systemic ACT treatment promotes psychological flexibility through various procedures, characterized by using experiential and attentional exercises (mindfulness), metaphors, clarification of values and the undertaking of actions committed to those values. ACT targets psychological flexibility processes by strengthening the following six core skills: acceptance (experiencing both pleasant and unpleasant thoughts, feelings, memories and impulses instead of trying to control or avoid them), contact with the present moment (consciously engaging in any moment, being mindful of thoughts, feelings, bodily sensations, and potential actions, even during distressing experiences), defusion (stepping back and observation of ideas as a representation of thought processes, seeing them for what they are and not as literal truths), self-as-context (developing a broad perspective on thinking and feeling and a nonevaluative, observing self), values (clarifying and defining fundamental values and goals to pursue meaningful work), and committed action (taking effective action guided by values; Hughes et al., 2017). ACT aims to help individuals with depression reduce mental rigidity, increase psychological flexibility, and adopt behaviors consistent with their values (Hayes et al., 2011).

ACT has gained more and more empirical support in treating depression (Twohig and Levin, 2017; A-Tjak et al., 2018). Studies have shown that ACT significantly improved participants’ psychological flexibility and emotional regulation and ultimately reduced their depressive symptoms (Zemestani and Mozaffari, 2020). ACT is an effective treatment for depression when assessed in individual, self-help, and group formats (A-Tjak et al., 2018; Kyllönen et al., 2018). A recent meta-analysis showed that ACT significantly reduced depressive symptoms compared to controls (Bai et al., 2020).

While these quantitative findings are essential, more research is needed to know how patients perceive treatment and the factors that influence the treatment effect. Patients’ treatment experiences can be analyzed qualitatively to improve treatment approaches and outcomes (Robert, 2008). Interpretative Phenomenological Analysis (IPA) is a qualitative approach to the study of how people make sense of their lived experiences, with a particular focus on those experiences that are significant and particular to the individual (Smith et al., 2009). Some researchers used IPA to examine the effects of short-term psychoanalytic psychotherapy (STPP) on the major depressive disorder in adolescents (Housby et al., 2021). IPA was proposed by Smith and is based on phenomenological, hermeneutic, and case study theories, suggesting that it is most appropriate when one is trying to explore how individuals perceive the particular situations they face and how they interpret their world and society, especially focusing on the complex journeys of their cognitive, linguistic, emotional, and physical states (Larkin and Thompson, 2011). Accordingly, IPA is an appropriate approach to explore the therapeutic experience of ACT for adolescents with depression. However, no qualitative studies have been conducted on adolescents with depression treated with ACT. This study aims to fill this gap by investigating the treatment experience of adolescents with depression. Therefore, the primary focus of this study is to explore how adolescents with depression improve through ACT, by increasing knowledge of how treatment works, with the aim of better understanding the mechanisms that may lead to positive changes in ACT treatment and providing a basis for improving clinical outcomes of ACT.

## Methods

### Design

This was a qualitative study. Adolescent patients aged 12–18 years in the psychotherapy clinic of a mental health center in Shandong Province were recruited for the study. According to IPA recommendations, a sample size of 4–6 is recommended to adequately investigate each participant’s experience and to provide some cross-case comparisons (Smith et al., 2009). In this study, a sample of five case interviews with good treatment outcomes in the act system was purposefully selected by the study objectives and empirical principles to gain an in-depth interpretive understanding completely and accurately. All of them received systematic ACT therapy 20 times based on voluntary participation. The therapeutic experience is then analyzed through semi-structured qualitative interviews, systematic text enrichment, and phenomenological interpretation.

### Data collection

In-depth interviews were primarily used to understand the interviewees’ experiences of managing their depressive symptoms through ACT treatment. Before the interview, the researcher prepared a tape recorder, an interview informed consent form, and an interview outline. Before the interview, the interviewer completed three main tasks: self-introduction and relationship building, explaining the purpose and procedures of the interview, and signing the informed consent form, emphasizing the maintenance of the interviewee’s interests and the principle of confidentiality.

Semi-structured interviews require the researcher to design an interview outline as the outline and basic framework of the interview according to the questions and purposes of the study before the interview is conducted, and to effectively collect the information and data needed for the study by talking with the interviewees. In our study, the interview outline included the following. (1) adolescents’ impressions of how things changed after being sent to the psychotherapy room; (2) their experiences in therapy; and (3) adolescents were also allowed to describe their therapy “stories” in the interview, which included some investigation of their interactions with the therapist.

In qualitative research, the researcher himself or herself is the research instrument. The researcher’s professional background, gender, age, personal abilities, personality traits, past research experiences, attitudes and experiences will affect the validity of the study. The researcher herself needs to be reflective and aware of her own identity at all times: the interviewer in this paper is a professional worker and researcher in clinical psychology with systematic training in counseling and psychotherapy. The semi-structured interviews were conducted face-to-face in a psychotherapy room, and each interview lasted about 60–90 min. During the interview, the interviewer could flexibly handle the interview questions according to the actual situation, and was not limited to the interview sequence of the outline, but changed the focus and questions of the interview according to the specific interviewees and the progress of the interview, depending on the time and place. The interview outline was only used as a prompt to follow the interviewee’s emotions and stream of consciousness and to follow up and interact with the interviewee at the right time to understand more about the interviewee’s inner experience. During the interview, the researcher began to hear the same conversation over and over again, indicating that data saturation had been reached and that it was time to stop collecting data and begin work on data analysis.

### Participants

Table 1 shows that the participants ranged from 12 to 18 years old (mean = 15.28, SD = 1.40). They were chosen based on the following criteria: (1) They received systematic ACT treatment; (2) They had a successful treatment outcome, as measured by the fact that they no longer met the HAMD (Hamilton Depression Scale) (Hamilton, 1960) diagnostic criteria for major depression at the end of treatment (20 weeks), and their self-reported MFQ (Mood and Feeling Questionnaire) (Angold and Costello, 1987) decreased by at least 5 points between baseline and end of treatment on the MFQ, which is considered to be the slightest clinically significant difference (Goodyer et al., 2011). Exclusion criteria: (1) adolescent patients who lacked the willingness to participate in the study; (2) concurrently receiving other genres of psychotherapy.

**Table 1:** Participant demographics.

Participant	Gender	Age	Grade	Sessions attended	MFQ score at baseline [T1]	MFQ score at the end of therapy [T2]
01	Female	13.6	Junior 1	20	54	22
02	Female	15.2	Junior 2	20	59	23
03	Male	17.4	Senior 2	20	54	23
04	Female	14.6	Junior 2	20	42	21
05	Male	15.6	Junior 3	20	40	21

### Ethics approval and consent to participate

Ethics approval (project ID: 2022YX043) has been granted by Weifang Medical University Ethics Committee. Participants and their legal guardians gave verbal and electronic informed consent to participate in this study.

### Analysis

The data were analyzed using interpretive phenomenology. IPA is a recently developed qualitative study of psychology. Qualitative data analysis followed IPA standards. This study follows the three theoretical principles of IPA (phenomenology, hermeneutics, and hagiology), the research process is clear and transparent enough, reasonably organized, and each topic is supported by sufficient evidence.

First, the interviews were transcribed, and the first author read each transcript several times. First, the interviews were transcribed, and the first author read each transcript multiple times. During the multiple readings, notes were made in the transcripts, including exploratory comments, to understand the participants’ experiences of managing depression. Second, the notes taken were turned into emergent themes following a thorough inspection of the individual transcripts to develop exact phrases at a higher level of abstraction to reflect the material. Third, emerging topics were categorized based on conceptual similarities, generating clusters. A descriptive label was assigned to each theme. The study team was briefed on these. In practice, each case’s text was aggregated and differentially examined first, and then the text was aggregated and differentially analyzed again. Smith et al. (2009) recommend that researchers determine how many participants an urgent theme or superordinate theme must be applied to be included in the final theme structure at this stage. It varies depending on the project’s purpose, with recommendations ranging from one-third to all participants. This study aimed to determine what factors contribute to improvement in the ACT treatment modality. Therefore, the researcher deemed the themes retained to be appropriate in the accounts of most participants. At the same time, themes that were not salient to the research question or did not apply to at least four participants were excluded. Finally, the themes were further evaluated and grouped to provide a final list of upper-and lower-level themes representing the participants’ everyday experiences.

### Results

**Figure 1:**
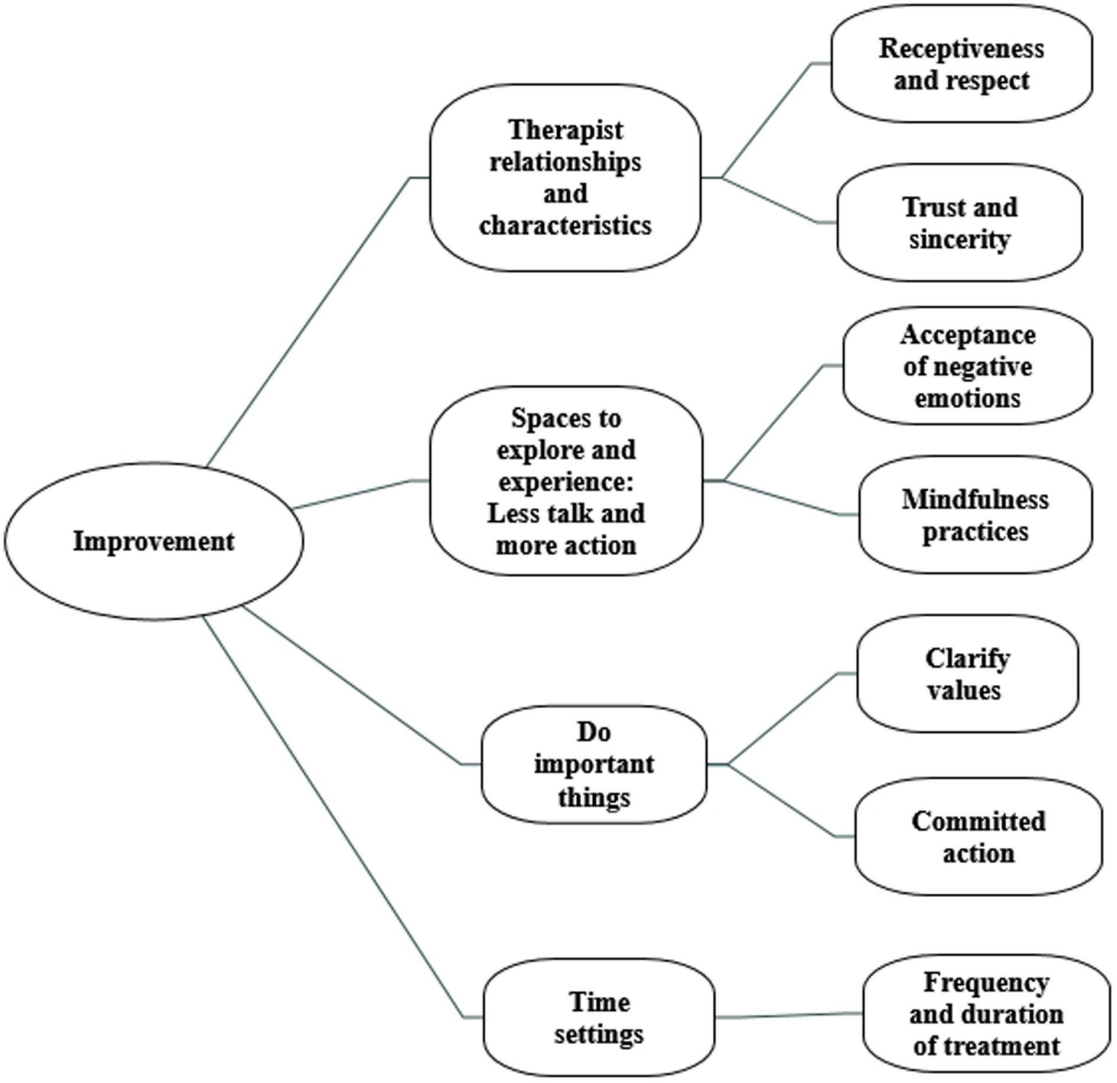
The figure shows the different themes and main categories that experienced an improvement in ACT for adolescents.

Adolescents experience several factors contributing to treatment improvement, and their experience under ACT conditions is summarized into four main themes, as shown in Figure 1. This section describes each topic and provides data to support analytical interpretation. “Therapist relationships and characteristics” represent receptiveness and respect from the therapist and having a trustworthy and sincere therapist. “Spaces to explore and experience” describes the ongoing process and content of acceptance of negative emotions and mindfulness practices in the healing process. “Do important things” represents values and committed action. “Time settings” describe the treatment frequency and duration (see Figure 1).

### Theme 1: Therapist relationships and characteristics

All treatment models agree that it is essential to establish a good therapist relationship, especially for the ACT. Almost all of the teens talked about the importance of therapeutic relationships.

### Receptiveness and respect

Warm, mutually cooperative relationships are naturally established when the therapist is fully present with the adolescent depression, receiving any emotional state that arises, dissociating from their judgments, and connecting with corresponding core values such as relationships. One adolescent said, “In the therapist’s presence, I felt I could speak about anything without worrying about how she was judging me, let alone trying to play up some supposedly good status deliberately.” Another patient said, “When talking to the psychotherapist, I can express any emotional feelings and thoughts about myself as much as I want because she always receives these emotions and thoughts, and this receptiveness from the therapist makes me feel understood, and it feels great.”

The therapist brings receptiveness, compassion, and curiosity to the visitor with full attention, which is therapeutic. “No one has ever listened to me so intensely as she did, without randomly interrupting me or judging me for what I said or did, and her eyes made me feel warm and safe.” When describing how therapy contributed to improvement during the session, one patient said it this way, “Instead of being impatient with me, the therapist sat steady, and when she came to talk to me with a kind, receptive look, with a caring, relatively calm tone of voice, I found it helpful.”

The adolescent paid close attention to the therapist’s respect and receptiveness to the adolescent, as reflected in their words and actions during the therapy session. As one adolescent said, “The therapist always asks for my opinion before each experience or exercise and never forces me to do it. “Another patient described it this way, “This receptiveness from the therapist would make me feel more and more stable, and this receptiveness was very powerful, and it was based on a profound understanding of me.” One patient said, “The feeling I get from the therapist is in stark contrast to the feeling I get from others around me. The family, relatives, and friends around me would always create a feeling in me that was so bad that it would make me feel wrong, negative, and always denied, and it played out like a terrible spell that cycled through my life.”

### Trust and sincerity

Sincerity means the therapist is honest and open with the visitor without deception, pretense, or evasion. One teenager put it this way: “When the therapist was very sincere in expressing negative experiences and feelings about herself, I felt that she was sincere and not false, not pretending to be herself because of her role as a therapist, which made me trust her more. When I knew that we all need to overcome the same struggles that we encounter in life, including disappointment, rejection, failure, anxiety, and insecurity, I found myself much more relaxed at that moment when I felt that we were comrades in the same boat, easily trapped in our thoughts.”

One of the adolescents had a previous unsuccessful experience with another service where he felt that the therapist was always giving him a ‘superior’ attitude, which made him unable to reveal his true self. As a result, he found it helpful to have an honest and trustworthy relationship in therapy.

Another patient described: “When the therapist asked, ‘Is it okay if we try this together, it was as if I was in cahoots with the therapist. This expression of hers made me feel that the therapist was helping me, was with me, was an invitation to me, not a correction or an order, so this part also triggered my motivation to want still to come next time.” One adolescent said, “I trust my therapist, which is essential to me, both in terms of her professionalism and character, and this trust makes me more motivated to engage in therapy.”

### Theme 2: Spaces to explore and experience: Less talk and more action

This second theme describes the adolescents’ experiences of the sessions themselves. ACT is a therapy with a strong emphasis on action. The therapist leads the visitors through many experiential exercises that range from a dozen seconds to half an hour. When discussing how therapy has helped them, most visitors talked about the importance of sticking with the various experiences and exercises. One adolescent said, “I often receive touches and inspiration during some of the experiences and exercise, which changed me a lot.” Another patient said, “Understanding the principles of therapy and experiencing the process are two completely different things, so practice is crucial, and more action is helpful to me.” When talking about how exercises have helped her, one adolescent described it this way, “The therapist would sometimes give me simple homework assignments, such as 20 sessions of positive breathing exercises, anchoring exercises, to transfer the experiences and exercises in the therapy room into my daily life, which I found very helpful.”

### Acceptance of negative emotions

Acceptance allows our thoughts and feelings to be as they are, whether pleasant or painful, to be open to them, to give up fighting them, and to allow them to be. Permit ourselves to have painful personal experiences if such experiences enable us to follow our values and approach unwanted personal experiences with a complete, open, unguarded state of mind. One teenager said: “It is not about liking or wanting them. It is about making room for them and observing your feelings like a curious scientist.” Another patient said: “I will not waste my energy trying to get rid of them. Miraculously, I noticed they softened when I gave them some space.”

One patient had powerful anger toward herself, and the therapist guided her to become aware of her feelings and then visualized them. He described it this way: “The therapist asked me to feel the color and temperature of the emotion and then rate the intensity of my emotion. I felt that the intensity of the anger decreased, so I thought it was helpful.”

Another patient said: “When my therapist asked me to try to normalize my feelings by evaluating them on a scale of 1–10 and naming them, I found my strained body beginning to relax. “.

### Mindfulness practices

Mindfulness means stepping back from the past or a constructed future and focusing on the present nonjudgmentally. It enhances our conscious awareness of the present moment, enabling us to grasp precisely what is happening at the moment, gather essential information to decide whether to change or persist in the behavior and thus help us to fully engage in what we are doing, increasing our sense of efficacy and satisfaction.

One adolescent described it this way: “The therapist told me to put my feet firmly on the floor, pay attention to the ground under my feet, and how I was sitting. I remember that was the first time I noticed that the pot of greenery on the table was that bright green color, and as a curious scientist, I looked at it closely and found that one of the greenery leaves was covered with yellow dots. It turns out that when we pay attention, life becomes much richer than what we take for granted in our minds.”

One patient said, “Pay attention to your breath, know that you are inhaling when you inhale, know that you are exhaling when you exhale and be aware, observe, and experience changes in your body’s response. Through the practice of watching the breath, observing the body, externalizing it, visualizing it, and keeping myself away from it, it is still there, not gone, but becoming less of an influence on me. But I can jump out, several times with the practice of breathing, and imagine myself pulling away.” Another teenager said, “When I am in a certain situation, I try to experience what my body is feeling and how it feels. Through this practice, I try to be with my emotions.”

One patient said how the practice of mindfulness practices had helped her: “I find it a seemingly simple but difficult thing to pay attention to my breath, always getting carried away by what is going on inside my head, but by sticking with it, I find that a certain moment frees me from the endless thoughts and becomes slightly calmer because the breath is always in me, the breath is always in the present moment. Practicing positive breathing for 10–20 min daily is very helpful.”

## Theme 3: Do important things

### Clarify values: Know what is essential

Values are statements about what we want to do in life, what we want to believe, and how we want to behave on an ongoing basis. They are the guiding principles that guide and inspire us forward in life. This outcome motivates everything we do in the ACT field: we do not want someone to accept pain, practice defusion, or expose themselves to challenging situations unless that behavior makes their life more prosperous or more fulfilling.

One teenager with severe test anxiety that he felt his life had no meaning said through values clarification during therapy, “Maybe getting into a good high school is not the end. Being a hard worker is the meaning I seek.”

One teenager said, “To be loved and respected by others, I want to strive to be a person who makes myself loving and respectable. There are many things I can do in this direction.”

One patient said, “It finally became clear to me that what I needed to do most was act courageously rather than reduce my feelings of anxiety. Understanding this has made me feel like I have a choice about my life.”

One teenager, who was always influenced by her teachers’ behavior and liked to judge them, said, after clarifying her values, “I want to become a person who learns to be accepting, understanding and appreciative.”

One patient chose the value of solidarity and closeness with her family, and she was particularly concerned about correspondence with her mother, saying, “I want to adopt behaviors that are consistent with the direction of the value–to increase support for my family and to reduce harm to my family, any time I can act in that direction.”

### Committed action

Committed action means adopting increasingly effective patterns of behavior guided and facilitated by values; it also means acting flexibly. Every therapy session requires committed action. The therapy is committed to action, conducting mindfulness practices or discussing topics about suffering and doing ACT homework. One teenager said, “When we commit to practice by turning our values into concrete goals, the process is not always smooth, and there are always psychological obstacles.” One patient said, “Change usually brings discomfort, usually anxiety. We must accept that discomfort and move on rather than stay in our comfort zone.”

When we want to change, our brain generates negative thoughts such as I’m depressed, I cannot do it, it is too difficult, there is not enough time, and so on. Then you can practice defusion exercises. One teenager said, “Thank the brain, good reason to give.”

Once an area of life and some core values are identified, learning to take small steps is important; as one teen put it this way, “Get clear on what your small step forward is going to be in the future and make sure it is the easiest and most likely to be done in the next 24 h.”

One patient said, “The therapist asks me to say my commitments aloud and accurately, which is a little shy and uncomfortable, but makes sure I can make room for those thoughts and feelings.”

## Theme 4:Time settings

The time setting refers to the frequency of the sessions and duration of the sessions. For different reasons, adolescents expressed satisfaction with the duration of treatment, which was initially 20 weeks. One patient talked about the adolescent’s unique condition and how they would be treated. Because of the complex changes in her life, she was allowed to continue her effective treatment after the first 20 weeks: “If the treatment had been stopped at the end of the session, I believe I would have felt empty inside, and in my situation at the time, I would have been very uncomfortable with the changes.” “The ability to receive a lengthy enough therapy period was quite important for me,” another added, “since I do not think a short-term treatment would have allowed me to do much.” One adolescent mentioned, “The weekly time made aside for continuous treatment has been critical to my progress in therapy, and I believe it is just as important as the therapy itself. Throughout the process, I always sense the therapist’s support and companionship, and that support and companionship are consistent and empowering.” Another patient discussed the advantages of having weekly appointments for that therapy “It is beneficial to have a clear plan to maintain a consistent time each week. It is a fantastic method to make daily living easier.”

## Discussion

The study revealed four themes important to adolescent progress: “Therapist Relationships and Characteristics,” “Spaces to explore and experience,” “Do important things,” and “Time setting.” Our findings are consistent with previous studies of ACT interventions (Østergaard et al., 2020). Patients experienced improvement through extensive exploration and experience (acceptance of negative emotions and mindfulness practices) and building a relationship with the therapist (therapist relationships and characteristics). Doing essential things (values and committed action) motivates patients to change. Patients acquire more appropriate internal working patterns and see progress over time (time setting). These factors should be considered when treating adolescent depression. As seen in Figure 1, the four themes are made up of dynamic elements. We refer to them as “improvement” and discuss some key components below.

The first theme emphasizes the critical role of the therapeutic relationship in treatment. First, therapists help depressed teens by communicating receptiveness and respect. This study’s findings are consistent with prior research (McCann et al., 2012; Binder et al., 2016; Jones et al., 2017). Therapist listening and support are crucial factors in teenagers’ willingness to participate in therapeutic procedures (Wilmots et al., 2020). Adolescents see therapists as “friendly” persons, and therapists build an equal and cooperative connection with them through respect and receptiveness. When therapists take an “expert” stance, making assumptions without genuinely listening to the experiences of adolescents, this attitude is viewed as disrespectful and interferes with the development of effective therapeutic connections. Adolescents who felt heard and could talk about anything in a nonjudgmental atmosphere indicated a level of freedom they did not have access to in other support networks in this study. It helps depressed teenagers create solid therapeutic relationships and boosts their motivation to overcome obstacles. This key point must be considered by therapists treating adolescent depression.

Adolescents are also more likely to participate in the treatment process and receive therapeutic experience if they see therapy as a collaborative effort and believe their therapist is a genuine and trustworthy individual who cares about their emotional and psychological well-being (Marotti et al., 2020). The above findings are consistent with this study’s findings in creating positive change. This finding could be because adolescents are in a unique period of transition from childhood to adulthood, with a focus on autonomous decision-making in collaboration (Noyce and Simpson, 2018), and therapists’ sincerity and trust can help improve adolescent depression patients’ treatment adherence and willingness to cooperate, as well as promote the development of their self-awareness. There will be a positive change.

The second theme describes the adolescents’ experiences in these programs. According to this research, acceptance of negative emotions is critical in treating depression. Research has found that experience avoidance has been identified as an essential contributing factor to depression (Shallcross et al., 2010; Rueda and Valls, 2016). Empirical avoidance refers to the suppression or avoidance of unwanted “personal experiences,” usually in rumination, in which people deliberate (Berzonsky and Kinney, 2019), while acceptance is an alternative to experiential avoidance. Acceptance is giving up the struggle for inner thoughts and exploring inner feelings and thoughts, whether pleasant or unpleasant, in a positive and curious state of mind (Hayes et al., 2012). It is an action taken on inner experiences (Twohig and Levin, 2017). In these ACT cases where the treatment is good, the therapist guides them to re-recognize the negative emotions; trying to eliminate all negative feelings and thoughts is impossible. It may be a wrong and ineffective direction. Conversely, when depressed adolescents stop trying to fight and eliminate these negative emotions and allow them to exist naturally, positive Change occurs, which is generally thought to help improve the effectiveness of psychotherapy.

Mindfulness is the ability to take a step back from the past or an imagined future and focus on the present moment nonjudgmentally (Zettle, 2007). This study backs up previous findings, and clinical investigations have shown that Mindfulness-Based Stress Reduction can lower emotional discomfort and stress, which is good for mental health (Díaz González et al., 2018). Studies have discovered mindfulness mediates depression outcomes (Kuyken et al., 2010; Spijkerman et al., 2016); Preventing lapses into verbally manufactured judgments, appraisals, and comparisons (Luoma and Villatte, 2012) requires awareness of moment-to-moment sensory and physical experiences. Therapists employed mindfulness practices to teach depression-stricken teenagers how to interact with the present moment in a focused, spontaneous, and flexible manner, allowing them to respond more flexibly and meaningfully to internal and external obstacles.

The third theme highlights the vital role that values and commitment play in the healing process. Loss of value has been highlighted as a fundamental component of the development of depression, according to Hayes et al. (2012) Ratcliffe (2012), Frankl (2014), Chan et al. (2020). A link between values and psychological suffering has been discovered in numerous studies (Reilly et al., 2018; Grégoire et al., 2021). As a result, the goal of this study is to encourage adolescents to take more explicit value-based acts. It has been discovered that values-oriented exposure allows the client to truly experience the meaning and effect of the treatment-induced discomfort. ACT’s capacity to make significant progress in the action stage is based on helping clients articulate their true beliefs. This aspect is something that therapists who work with depressed teens should consider.

The fourth theme highlights the element of time setting. Time was linked to the experience of getting a sufficient number of therapy sessions as a helpful setting, and all five patients in this study had had 20 sessions. The improvement was aided by fixed weekly visits with the therapist instead of irregular appointments. Adjusting the frequency and duration of treatment cycles to meet the changing needs of adolescents’ daily lives can help improve. This finding is consistent with the findings of Gibson et al. (Gibson and Cartwright, 2013). Adolescents appear to experience a possibility of maturation and growth with the vital aid of their therapist over time, during, and between therapy sessions. Well-established therapy period can provide the steadiness that an adolescent needs amid the chaotic and stressful stages of their life.

### Strengths, limitations, and future research

The main strength of this study is the in-depth focus on the experiences of those who engage in ACT, as no studies have examined this experience to date. The specific methodology of IPA means that participants who express less about their therapy experience are equally represented in the data, which provides a rich representation of convergence and divergence in their backgrounds that is not possible using other methods. However, caution should be exercised when considering how the results transfer to the experiences of other adolescents, whether in impact studies or different Settings. The fact that these therapies are offered as part of clinical trials also limits the transferability of research results. The sample included adolescents interviewed as part of routine clinical psychotherapy practice, meaning their particular experiences might set them apart. Because our selection had only those who did “well” on treatment, it was impossible to extrapolate the experiences of those who did less well. Because the sample population is concentrated in Weifang, Shandong Province, promotion to the broader international population may also be limited.

In addition, with the development of the Internet and communication technology, the combination of ACT with low-cost, easily accessible self-help treatment is one of the directions for future research.

## Conclusion

The study examined the experiences of five adolescents with depression in improving ACT. With a therapist who is receptive and respectful of adolescent patients, they make positive changes by exploring themselves in a sincere and trusting therapeutic relationship. Progress seems to come through being open to all thoughts and feelings and developing the ability to live in the present moment. They attach great importance to value-oriented behavior, using core values to guide, motivate and encourage themselves to change their behavior. Furthermore, we think that is the critical thing that therapists need to pay attention to in the treatment of depression, and these themes help identify what it is about depressed adolescents that makes ACT effective in guiding them toward recovery.

## Data availability statement

The raw data supporting the conclusions of this article will be made available by the authors, without undue reservation.

## Ethics statement

Written informed consent was obtained from the individual (s), and minor (s)’ legal guardian/next of kin, for the publication of any potentially identifiable images or data included in this article.

## Author contributions

Interviews were conducted by JM alone. JM and LJ wrote the principal analysis and discussion. All authors completed the analysis and discussion and read and approved the final manuscript.

## Funding

The work was supported by Natural Science Foundation of Shandong Province (ZR2016GM05) supported the research.

## Conflict of interest

The authors declare that the research was conducted in the absence of any commercial or financial relationships that could be construed as a potential conflict of interest.

## Publisher’s note

All claims expressed in this article are solely those of the authors and do not necessarily represent those of their affiliated organizations, or those of the publisher, the editors and the reviewers. Any product that may be evaluated in this article, or claim that may be made by its manufacturer, is not guaranteed or endorsed by the publisher.

## Abbreviations

ACT, Acceptance and Commitment Therapy
CBT, Cognitive and Behavior Therapy
IPA, Interpretative Phenomenological Analysis
HAMD, Hamilton Depression Scale
MFQ, Mood and Feeling Questionnaire
